# Global Cognitive Impairment Prevalence and Incidence in Community Dwelling Older Adults—A Systematic Review

**DOI:** 10.3390/geriatrics5040084

**Published:** 2020-10-27

**Authors:** Ricardo Pais, Luís Ruano, Ofélia P. Carvalho, Henrique Barros

**Affiliations:** 1 EPIUnit—Instituto de Saúde Pública, Universidade do Porto, Rua das Taipas, n° 135, 4050-600 Porto, Portugal; lmruano@gmail.com (L.R.); opcarvalho20@gmail.com (O.P.C.); henrique.barros@ispup.up.pt (H.B.); 2 Departamento de Epidemiologia Clínica, Medicina Preditiva e Saúde Pública, Faculdade de Medicina da Universidade do Porto, Alameda Prof. Hernâni Monteiro, 4200-319 Porto, Portugal; 3 Unidade de Saúde Familiar Lusitana, Aces Dão Lafões, A.R.S. Centro, Av. António José Almeida, 3514-511 Viseu, Portugal; 4 Departamento de Neurologia, Hospital de São Sebastião, Centro Hospitalar de Entre o Douro e Vouga, Rua Cândido Pinho, 4520-211 Santa Maria da Feira, Portugal

**Keywords:** epidemiology, cognitive impairment, prevalence, incidence

## Abstract

(1) Background: We proposed to review worldwide estimates of cognitive impairment prevalence and incidence in adults older than 50 years of age living in the community. (2) Methods: Systematic searches were performed in January 2019 using MEDLINE/PubMed. Articles were selected if they referred to cognitive impairment, prevalence, incidence, elders, and population or community-based studies. Analysis, aggregated by different methodologic features, was performed. (3) Results: Prevalence (80 studies) ranged between 5.1% and 41% with a median of 19.0% (25th percentile = 12.0%; 75th percentile = 24.90%). Incidence (11 studies) ranged from 22 to 76.8 per 1000 person-years with a median of 53.97 per 1000 person-years (25th percentile = 39.0; 75th percentile = 68.19). No statistically significant effects were found except for inclusion age. (4) Conclusion: We propose that the homogenization and clarification of the definition of what constitutes cognitive impairment are essential to refine the epidemiological understanding of this entity. The results of this review reinforce the importance of adherence to standardized cut-off scores for cognitive tests to promote study comparability.

## 1. Introduction

The size of the elderly population is increasing worldwide. The United Nations project that this increase will intensify in the coming decades, mostly due to the rise in average life expectancy. The number of elderly people in the world (more than 60 years old) will increase by 56% in the next 15 years and the “oldest old” (more than 80 years old) will triple in number by 2050 [1]. This rapid demographic ageing will increase the prevalence of disease and disability, with a particular emphasis expected for the impairment of cognitive functions [2].

Loss of memory, learning difficulties and a decrease in the ability to concentrate on a task characterizes cognitive impairment in the elderly [3]. This ranges from mild deficits, which are not clinically detectable, to dementia [4]. There are many different etiologies of cognitive impairment, ranging from vascular conditions to neuronal degeneration and stroke. Cognitive impairment leads to a decrease in the life quality of elders and increases the risk of dementia and mortality [5,6]. Additionally, it has significant social consequences, resulting in the loss of autonomy and independence and leading to an increased need for permanent caregivers and assistance by health services [7,8].

There is a scarcity of studies reporting the prevalence of cognitive impairment at a given time point, as well as of the incidence of newly diagnosed cases. Both of these measures help to identify disease trends within a population, giving information not only on how common the condition is but also at what speed new cases are emerging. This information is essential to assess the overall burden of disease and to develop hypotheses regarding the causes and factors that increase the risk of disease. Good quality scientific data on cognitive impairment are needed, both to identify groups at risk of developing cognitive changes at an early stage and to identify the optimum time at which to implement preventive and corrective measures. A better understanding of cognitive impairment and its lifetime course is needed to define and implement strategies to both prevent initial cognitive impairment and either stop or delay its progression towards dementia once established. In 2015, the COSMIC studies (Cohort Studies of Memory in an International Consortium) was published, which used data from cohort studies in several countries around the world, applied uniform criteria to harmonize data, and reported the prevalence of cognitive impairment [9]. Our systematic review complements the COSMID study as it includes information on the prevalence as well as incidence of cognitive impairment by considering the latest studies published after 2015, and it includes information from Portugal.

The free-form research question we used to drive this research was “What is the worldwide cognitive impairment prevalence and incidence in older adults, as reported by observational studies?” The PICO structure to our research question was as follows: Population—older adults; Intervention—observational studies; Comparison—worldwide; Outcome—prevalence and incidence of cognitive impairment. Our objective was to review the global epidemiological data on cognitive impairment and to derive prevalence and incidence estimates for this nosological entity.

## 2. Materials and Methods

We conducted a systematic search of the PubMed electronic database on 4 January 2019. We did not seek unpublished data. We considered all studies published until 4th January 2019 for the analysis. The search details were “cognitive impairment”[All Fields] AND ((“epidemiology”[Subheading] OR “epidemiology”[All Fields] OR “prevalence”[All Fields] OR “prevalence”[MeSH Terms]) OR (“epidemiology”[Subheading] OR “epidemiology”[All Fields] OR “incidence”[All Fields] OR “incidence”[MeSH Terms])) AND (elders[All Fields] OR older[All Fields]). For the first evaluation, we imported a total of 3645 references to Endnote. In order to increase the information for Portugal, we conducted a second related search on the same day. The search details were “cognitive impairment”[All Fields] AND ((“epidemiology”[Subheading] OR “epidemiology”[All Fields] OR “prevalence”[All Fields] OR “prevalence”[MeSH Terms]) OR (“epidemiology”[Subheading] OR “epidemiology”[All Fields] OR “incidence”[All Fields] OR “incidence”[MeSH Terms])) AND (elders[All Fields] OR older[All Fields] OR (“aged”[MeSH Terms] OR “aged”[All Fields])) AND (“portugal”[MeSH Terms] OR “portugal”[All Fields]). A total of 53 references were imported and added to the database. We did not limit the search results by the language of publication. We eliminated duplicates (8 references).

References were verified using a two-step process. For the first step, articles were selected based on information available in the title and/or abstract. The full text of the selected articles was read in the second step to determine the agreement of each article with the adopted criteria. We included reports with epidemiological data on cognitive impairment (CI), mild cognitive impairment (MCI) and cognitive impairment not dementia (CIND). These terms are used differentially but overlap to some extent and there was no standard rule that would allow us to draw a clear distinction between them, therefore they were assumed to refer broadly to the same entity and treated as such.

The exclusion criteria were as follows: non-original full-length articles (e.g., a systematic review, guidelines, meta-analysis, review, comment, editorial, note, meeting abstract); case-reports; non-human/in vitro; non-elderly population (studies conducted in populations described as consisting exclusively or partially of children, adolescents or adults); language (papers not written in English, Spanish, French or Portuguese were excluded); treatment/intervention/diagnostic studies; no data on cognitive impairment (studies that did not report prevalence or incidence of cognitive impairment); cognitive impairment in specific subgroups, such as patients with dementia, depression, HIV and Parkinson’s disease; studies including the oldest old only (over 85 years old); institutionalized participants in hospitals, clinics or nursing homes (to obtain data for older people present in the general population and not report on a special population).

We collected data regarding the participants’ age, sample size, diagnostic methods used, world region, and estimates of prevalence and/or incidence of cognitive impairment. We provide Supplementary Material with the characteristics of the cohort studies.

We assessed the quality of the studies included using the National Heart, Lung, and Blood Institute (NHLBI) Quality Assessment Tool for Observational Cohort and Cross-Sectional Studies [10], categorized as >80% yes = “Good”, 60–80% yes = “Fair”, and <60% yes = “Poor” to assess the internal validity and risk of bias for each study and the overall quality. We took into account the PRISMA (Preferred Reporting Items for Systematic Reviews and Meta-Analyses) 2009 Checklist (Table S4) and the Quality Assessment tool from the NHLBI to verify methodological quality and the quality of the included studies (Table S3).

Tables with the results of the Quality Assessment Tool and the score from the PRISMA 2009 Checklist are available as Supplementary Material.

### 2.1. Statistical Analysis

The included studies differed in several parameters such as participant inclusion age, sample size, diagnostic methods used and world region (Europe, Asia, North America, South America and Australia). Due to the large variance of reported prevalence and incidence estimates, we divided data into more homogeneous groups, and within-group comparisons were made with Kruskal–Wallis for independent samples test and the median quartiles using Tukey’s Hinges method. We used non-parametric statistical techniques due to the asymmetrical distribution of the sample. The statistical analyses were performed using SPSS**^®^** version 21. Prevalence was reported as a percentage and incidence is reported in cases per 1000 person-years, while the median (25–75 percentile) are reported for both.

### 2.2. Data Analysis

For prevalence data, we subdivided papers into three groups according to inclusion age: (1) participants aged from 50 to 59 years (mean = 52.93 years; SD = 2.50 years)—14 papers; (2) participants aged from 60 to 69 years old (mean = 63.28 years; SD = 2.49 years)—57 papers; and (3) participants aged 70 years or older (mean = 75.11 years; SD = 3.02 years)—9 papers. Regarding sample size, 26 studies had fewer than 1000 participants (mean = 504.19 participants; SD = 222.12 participants); 22 had between 1001 and 2500 participants (mean = 1695.82 participants; SD = 408.78 participants); 18 had between 2501 and 5000 participants (mean = 3614.33 participants; SD = 519.19 participants) and 14 studies had more than 5000 participants (mean = 7314.50 participants; SD = 1682.56 participants). According to the diagnostic method used to identify cognitive impairment, 9 studies accounted for the presence of cognitive complaints by either the patient or family, the absence of dementia and a neurological evaluation; 62 used only standard neurological tests to determine cognitive impairment (including but not restricted to Mini Mental State Examination (MMSE), Montreal Cognitive Assessment (MOCA), and the Short Portable Mental Status Questionnaire); and 8 simultaneously used both of the previously described methods. We analyzed data by world region: 25 studies in Europe; 13 studies in North America; 3 studies in South America; 35 studies in Asia; 2 studies in Africa; and 2 studies in Australia (Table S1).

To estimate the incidence of cognitive impairment, we divided the papers according to the same criteria: people aged: 50–59 years (mean = 55.33 years; SD = 0.58 years)—3 papers; 60–69 years old (mean = 64.50 years; SD = 2.38 years)—4 papers; ≥70 years (mean = 73.75 years; SD = 2.87 years)—4 papers. In terms of sample size, 2 studies had fewer than 1000 participants (mean = 608.50 participants; SD = 215.60 participants); 5 studies had 1001–2500 participants (mean = 1701.80 participants; SD = 479.51 participants); 3 studies had 2501–5000 participants (mean = 3102 participants; SD = 698.69 participants); and one study included 7166 participants. As for the diagnostic method used to identify cognitive impairment, three studies accounted for the presence of a patient or family report of cognitive complaints, the absence of dementia and a neurological evaluation; five studies used validated neurological tests; and three studies used both of the previously described methods. There were five studies carried out in both Europe and North America, and one was carried out in Asia (Table S2).

## 3. Results

**Figure 1:**
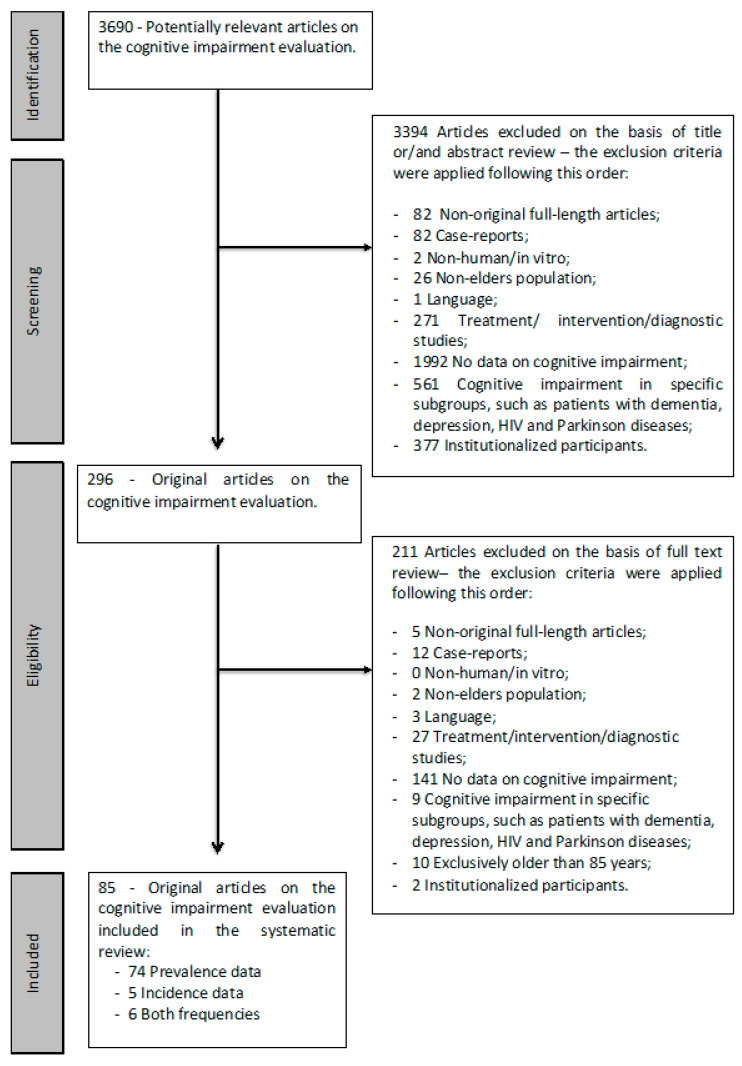
Flow chart summary of the literature search.

Of the 3690 potentially relevant articles found, 296 were selected based on the information present in the title and/or abstract (step 1); after reading the full text, 85 were selected as relevant (step 2). Of these, 74 papers provided information only on cognitive impairment prevalence, 5 papers only provided information on cognitive impairment incidence and 6 papers provided information for both parameters (Figure 1). The quality assessment tool from the NHLBI was used to assess the methodological quality of the included studies; 77 studies had an overall rating of “good”, eight were rated “fair” and none were rated “poor”. Based on these findings, no papers were excluded from the analysis.

### 3.1. Prevalence of Cognitive Impairment

**Figure 2:**
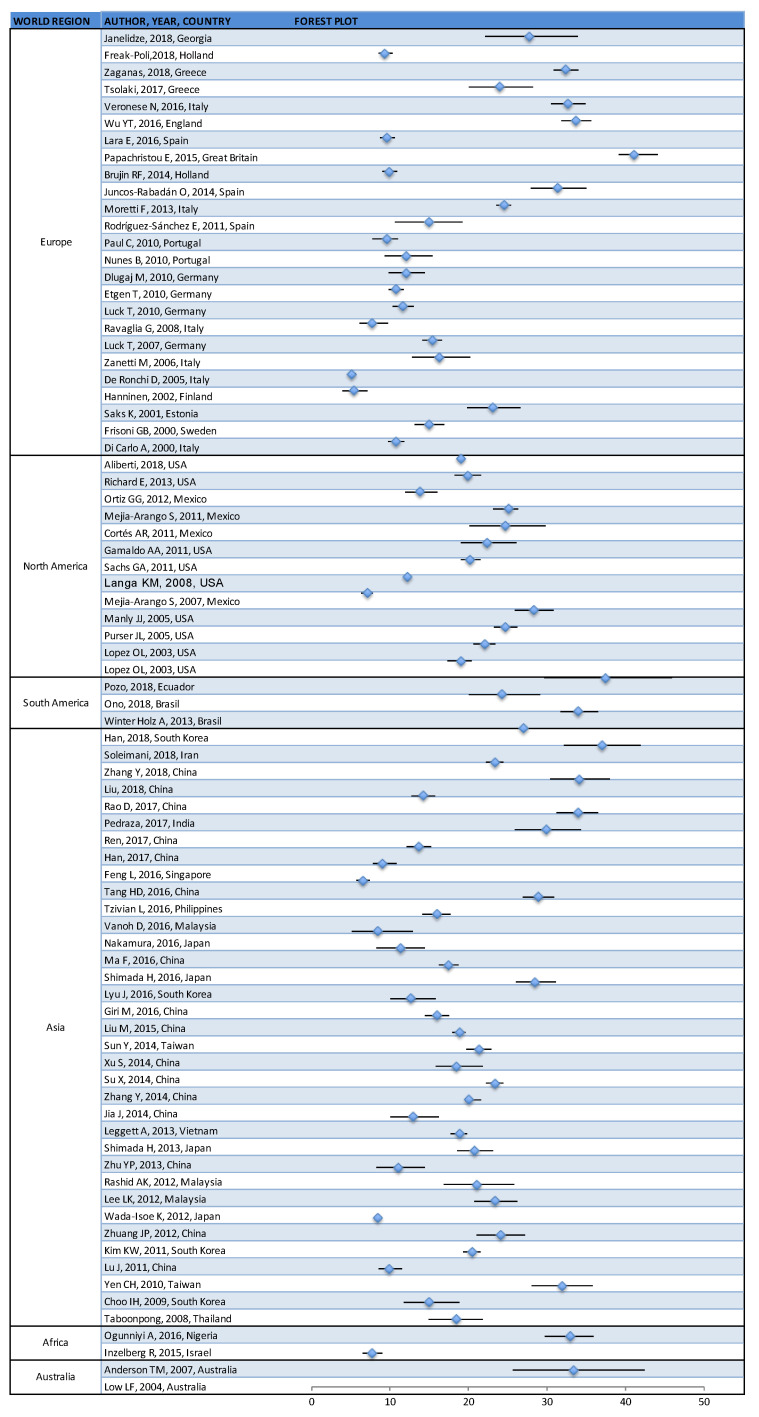
Prevalence of cognitive impairment reported by published papers, which are grouped by world region (95% confidence intervals were obtained from papers or calculated from the data presented).

The prevalence of cognitive impairment (CI) reported in the 80 studies [11,12,13,14,15,16,17,18,19,20,21,22,23,24,25,26,27,28,29,30,31,32,33,34,35,36,37,38,39,40,41,42,43,44,45,46,47,48,49,50,51,52,53,54,55,56,57,58,59,60,61,62,63,64,65,66,67,68,69,70,71,72,73,74,75,76,77,78,79,80,81,82,83,84,85,86,87,88,89,90] ranged from 5.1% to 41.0% (median = 19.0%; 25th percentile = 12.0%; 75th percentile = 24.90%) (Table 1 and Figure 2).

Grouping papers according to inclusion age (50–59 years old, 60–69 year old, and ≥70 years old), the reported prevalence ranges from 6.5% to 34% (median = 12%; 25th percentile = 9.6%; 75th percentile = 17.65%) in the first group [17,19,20,23,24,25,26,41,57,60,61,62,75,81], 5.1% to 37.5% (median = 20.1%; 25th percentile = 14.2%; 75th percentile = 24.7%) in the second group [11,12,13,14,15,16,21,22,28,30,31,32,33,34,35,36,37,38,39,40,42,44,45,46,49,50,51,52,53,54,55,56,59,63,64,65,66,67,68,69,70,71,72,73,74,76,77,78,79,80,82,83,84,85,86,87,88,89] and from 11.6% to 41% (Med = 19%; 25th percentile = 15%; 75th percentile = 29.90%) in the last group [18,27,29,34,43,47,48,58,90].

When grouping and analyzing the effect of sample size, we divided the studies into four groups based on the number of participants they had (<1001, 1001–2500, 2501–5000 and >5000). The reported prevalence in the first group ranged from 5.3% to 37.5% (median = 22.75%; 25th percentile = 14.9%; 75th percentile = 31.4%) [11,14,20,22,24,30,32,33,40,41,49,53,55,58,64,68,72,75,78,79,80,85,86,87,88,90], from 7.7% to 41% in the second group (median = 15.95%; 25th percentile = 11.60%; 75th percentile = 28.50%) [16,18,19,23,27,28,34,37,38,45,50,51,56,57,59,60,62,63,67,69,77,84,89], from 6.5% to 32.7% in the third group (Med = 13.75%; 25th percentile = 9.60%; 75th percentile = 21.30%) [12,13,15,17,25,26,29,35,42,44,46,47,48,61,66,71,81], and from 5.1% to 27% (median = 20.24%; 25th percentile = 18.8%; 75th percentile = 24.1%) in the last group [21,31,36,39,43,54,65,70,73,74,76,82,83].

Regarding cognitive impairment diagnostic methods, in the presence of cognitive complaints, the absence of dementia and with a neurological evaluation, the prevalence of cognitive impairment (CI) was from 9.6% to 33% (median = 15.4%; 25th percentile = 11.3%; 75th percentile = 23.4%) [17,19,20,25,29,37,65,80,88]. When only standardized neurological tests were used (MMSE, MOCA, Short Portable Mental Questionnaire, etc.), the prevalence of CI ranged from 5.1% to 41% (median = 18.9%; 25th percentile = 12.2%; 75th percentile = 24.7%) [11,12,13,14,15,16,18,21,22,23,26,28,30,31,32,33,34,36,38,39,40,41,42,43,44,45,46,47,48,49,50,51,52,53,55,58,59,60,61,63,64,66,67,69,70,75,76,77,78,79,81,82,83,84,85,86,87,89,90]. When both methods (neurologist evaluation, patient or family complaints and standardized neurologic tests) were used, the estimated prevalence of CI ranged from 10.7% to 34% (median = 21.30%; 25th percentile = 12.0%; 75th percentile = 28.90%) [24,27,35,56,57,62,71,72,73,74].

With regard to the world region where data were collected, in Europe the prevalence of cognitive impairment ranges from 5.1% to 41% (median = 12.1%; 25th percentile = 9.94%; 75th percentile = 23.9%) [11,12,13,14,15,16,17,18,19,20,21,22,23,24,25,26,27,28,29,30,31,32,33,34,35]; in North America, it ranged from 7.1% to 28.3% (median = 20.1%; 25th percentile = 19%; 75th percentile = 24.70%) [36,37,38,39,40,41,42,43,44,45,46,47,48]; in South America, it ranged from 24.3% to 37.5% (median = 34%; 25th percentile = 29.15%; 75th percentile = 35.75%) [49,50,51]. In Asia the prevalence ranges from 6.5% to 37% (median = 19.44%; 25th percentile = 13.25%; 75th percentile = 25.55%) [52,53,54,55,56,57,58,59,60,61,62,63,64,65,66,67,68,69,70,71,72,73,74,75,76,77,78,79,80,81,82,83,84,85,86]. In Africa, CI prevalence ranged from 18.4% to 33% (median = 25.7%; 25th percentile = 18.4%; 75th percentile = 33%) [87,88] and in Australia from 7.7% to 33.3% (median = 20.5%; 25th percentile = 7.7%; 75th percentile = 33.3%) [89,90]. No statistically significant differences within groups were found in the reported CI prevalence when grouping papers according to any of these variables.

### 3.2. Incidence of Cognitive Impairment

**Figure 3:**
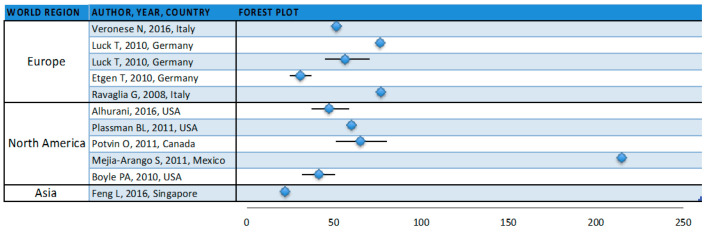
Incidence of cognitive impairment reported by the 11 included studies, which are grouped by world region (the 95% confidence intervals were obtained from papers or calculated with the data presented).

The incidence of cognitive impairment reported by the 11 included studies [15,26,28,39,60,91,92,93,94,95] ranged from 22 to 215 per 1000 person-years, with a median incidence of 56.50 per 1000 person-years (25th percentile = 41.77; 75th percentile = 76.50) (Table 2 and Figure 3).

Grouping papers according to the age of the participants (50–59 years old, 60–69 years old, and ≥70 years old) yielded incidence estimates ranging from 22 to 41.77 per 1000 person-years (median = 30.7 per 1000 person-years (25th percentile = 26.35; 75th percentile = 36.24) in the first group [26,60,95], from 51.45 to 215 per 1000 person-years (median = 71.11 per 1000 person-years (25th percentile = 58.44; 75th percentile = 145.98) in the second group [15,28,39,96] and from 47.19 to 76.50 per 1000 person-years (median = 58.45 per 1000 person-years (25th percentile = 51.84; 75th percentile = 68.45) in the last group [91,92,93,94]. Statistically significant differences were found in the incidence of CI across age categories (*p* = 0.035).

Taking into account the number of participants included in the studies (<1001, 1001–2500, 2501–5000 and >5000), the reported CI incidence ranged from 41.77 to 60.4 per 1000 person-years (median = 51.09 per 1000 person-years (25th percentile = 41.77; 75th percentile = 60.40) for group 1 [94,95], from 22 to 76.8 (median = 56.50 per 1000 person-years (25th percentile = 47.19; 75th percentile = 76.50) for group 2 [28,60,91,92,93], from 30.70 to 65.42 (median = 51.45 per 1000 person-years (25th percentile = 41.08; 75th percentile = 58.44) for group 3 [15,26,44,96] and the only study with more than 5000 participants reported an incidence of 215 cases per 1000 person-years. No statistically significant differences were found between the groups.

According to the cognitive impairment diagnostic methodology used, studies that evaluated the presence of cognitive complaints and the absence of dementia, and included a neurological evaluation, reported a CI incidence ranging from 41.77 to 215 per 1000 person-years (median = 76.5 per 1000 person-years (25th percentile = 59.14; 75th percentile = 145.75) [91,92,94]. The studies that used neurological tests (MMSE, MOCA, Short Portable Mental Questionnaire) reported an incidence from 22 to 76.80 per 1000 person-years (median = 51.45 per 1000 person-years (25th percentile = 30.7; 75th percentile = 60.4) [15,26,28,60,96]. The studies that used both methods reported a CI incidence ranging from 47.9 to 65.42 per 1000 person-years (median = 56.50 per 1000 person-years (25th percentile = 51.82; 75th percentile = 60.96) [39,93,95]. There were no statistically significant differences among these groups.

In Europe, the incidence of cognitive impairment ranges from 30.70 to 76.50 per 1000 person-years (median = 56.5 per 1000 person-years (25th percentile = 51.45; 75th percentile = 76.5) [15,26,28,91,92]. In North America, this ranged from 41.8 to 215 per 1000 person-years (median = 60.4 per 1000 person-years (25th percentile = 47.19; 75th percentile = 65.42) [39,93,94,95,96] and in Singapore the incidence was reported as 22 per 1000 person-years [60]. We did not find statistically significant differences among groups.

One study reported an incidence of 215 per 1000 person-years, which is 11.85 standard deviations over the mean of the other ten studies. Excluding that study from the data analysis changes the reported median incidence to 53.97 per 1000 person-years (25th percentile = 39.0; 75th percentile = 68.19). In the group of participants with the minimum inclusion age (60–69 years old), the median incidence was 65.42 per 1000 person-years (25th percentile = 58.44; 75th percentile = 71.11), statistically significant differences were found within the group (*p* = 0.05). In the group of studies that evaluated the presence of cognitive complaints, the absence of dementia included a neurological evaluation, the median incidence was 59.14 per 1000 person-years (25th percentile = 41.77; 75th percentile =76.50), and we did not find statistically significant differences within the group. In the group of studies from North America, the median incidence was 53.80 per 1000 person-years (25th percentile = 44.48; 75th percentile = 62.91), and we did not find statistically significant differences within the group.

## 4. Discussion

### 4.1. Methodological Considerations

Our objective was to review the global epidemiological data to derive prevalence and incidence estimates for cognitive impairment. We included reports with three different constructs: cognitive impairment (CI), mild cognitive impairment (MCI) and cognitive impairment not dementia (CIND). Besides the different names, we could not distinguish consistently between them, so all were assumed to refer broadly to the same entity and were treated as such.

We expected a significant degree of heterogeneity among studies, so data were aggregated by age group, study sample size, diagnostic methods used, and world region.

Despite all the studies having elderly people as the study focus, the minimum inclusion age for participants diverged greatly between studies and could bias the results. For example, higher estimates of cognitive impairment could be a result of a more elderly sample, as several different studies reported an increase in cognitive impairment prevalence with increasing age [17,20,45,92]. In our study, we found that in terms of the incidence of cognitive impairment, studies that had an inclusion age starting at 60 years had a median incidence higher than those with an inclusion age over 70 years old, and the difference was statistically significant. The lower incidence at higher ages might imply that the rate of conversion from healthy cognition to cognitive impairment might reach a plateau at some point after 60 but before 70 years of age, considering that cognitive impairment is a milder form of decline that, at older ages, can progress to dementia.

Regarding the sample size of the studies, the main objective was to compare the results of studies with hundreds of participants to others with thousands of participants, and we found no significant quantitative differences among these.

While there were no statistically significant differences regarding the method used to identify cases of cognitive impairment, for studies of the prevalence of cognitive impairment, the median prevalence of cognitive impairment was higher for the method that used a neurological evaluation paired with neurological tests. With regard to studies on the incidence of cognitive impairment, the median was higher for neurological evaluations. In the future, we aim to further explore the optimum methods to identify cognitive impairment and develop recommendations that will lead to a better and more accurate diagnosis [97].

We aggregated data by world region to examine the geographic differences that may influence the epidemiology. In terms of the prevalence of cognitive impairment, there were no statistically significant differences. However, in terms of incidence of cognitive impairment, in Europe it was lower than in North America; this could be due to cultural or genetic effects, or a combination of both, with an impact on cognitive impairment severity and progression [9].

Regarding cognitive impairment incidence studies, the Mejia-Arango [39] study from Mexico reports an incidence that is 11.85 standard deviations above the mean of the other ten studies (Figure 3). Although the median is not greatly affected by outliers, this study alone increases the median reported incidence from 53.97 per 1000 person-years (without) to 56.50 per 1000 person-years (with). Several procedural characteristics set this study apart. Briefly, it was the only study that used a version of the Cross-Cultural Cognitive Examination (CCCE) as a cognitive decline screening approach, which might point to a culturally diverse background of the participants. To those unable to complete the questionnaire due to limitations of language or health, a brief version of the informant questionnaire of Cognitive Decline in the Elderly (IQCODE) was applied. Additionally, 32.70% of the participants in the study were illiterate, and education years have a meaningful impact on cognitive impairment frequency [28,88]. The high cultural heterogeneity implicit in this choice of instruments and reported illiteracy prevalence raises doubts over whether this incidence estimate is valid for Mexicans in general or highly influenced by a specific sub-population within Mexico. Due to its methodological particularities and high incidence estimate, we analyzed all of the incidence variables excluding the Mejia-Arango paper; however, there were no statistically significant differences within groups with or without it.

### 4.2. Future Directions

The ability to rely on tests to identify the incipient cases of cognitive impairment with high accuracy (sensitivity and specificity) is crucial for population studies and population interventions, as it is both impractical and cost-prohibitive to have a specialist neurological assessment of large numbers of unaffected individuals. We believe that our results highlight the need for the development of a consensus regarding the best initial markers of cognitive impairment, the development of more reliable tests to detect incipient cognitive impairment cases, both with better reliability and more universally applied cut-off points that account for the factors known to influence cognitive declines such as age and education. Equally, to examine age-related cognitive decline, studies should restrict the inclusion age to 60 years old, as this is the threshold for older people as defined by the WHO [1]. We expect that the implementation of these measures would lead to a more reliable and valid diagnosis of cognitive impairment and a more accurate global view of the prevalence and incidence of cognitive impairment in older people, which is fundamental to the delineation of public health measures aimed at this risk group. Results from this systematic review may inform public health decisions through accurate regional estimates of cognitive impairment for the definition of adequate measures regarding modifiable risk factors, particularly in people over 60 years old. Detection and treatment of diabetes and hypertension, reduction in levels of obesity, smoking cessation, increased physical activity, and better education should be public health priorities. We also provide some suggestions for methodologies on further cognitive impairment studies, as there are significantly different social and economic structures in different world regions, and even in different countries within the same world region, it would be essential to conduct studies aimed explicitly at understanding cognitive impairment in the specific region.

### 4.3. Strengths and Limitations

The strengths of this study were its global view of the epidemiological data and the use of studies which reported on the general population, while excluding those that reported on people within the healthcare system or with a diagnosed underlying disease etiology. There are some methodological limitations and a risk of different types of bias associated with this study. Among these, we should mention publication bias, the selective reporting of data within studies and the incomplete retrieval of research. To try to reduce the risk of other biases, we aggregated papers into more methodologically homogeneous groups and compared the reported data within each group. By not restricting the initial search by publication language, we have an estimate of the size of our language bias. By only including reports written in Portuguese, English, Spanish or French, we excluded four studies. Reporting bias in published studies due to the selective reporting of subgroups of a population or the exclusion of non-significant outcomes measured by the study is a possibility that should be borne in mind. Additionally, we did not consider data on cognitive impairment etiology, as the main objective of this study was to review worldwide estimates of the prevalence and incidence of cognitive impairment in older adults regardless of etiology. Another limitation was that there was no pairwise review.

## 5. Conclusions

This systematic review reports that the global prevalence of cognitive impairment ranged from 5.1% to 41% with a median of 19.0%. The incidence of cognitive impairment ranged from 22 to 76.8 per 1000 person-years, with a median of 53.97 per 1000 person-years. We did not find statistically significant effects besides participant age in the studies sampled. For future studies, we propose the homogenization of the definition of cognitive impairment and the importance of the standardized cut-off scores of cognitive tests to compare different studies.

## Figures and Tables

**Table 1:** Summary of the prevalence of cognitive impairment reported by 80 studies included in analysis.

	Number of Papers	With Cognitive Impairment Median (25–75 Percentile)	*p* Value
Inclusion Age			
50–59	15	12.0 (9.60–17.65)	0.062
60–69	57	20.10 (14.20–24.70)	
≥70	9	19.0 (15.0–29.90)	
Participants Number			
<1001	26	22.75 (14.90–31.40)	0.386
1001–2500	22	15.95 (11.60–28.50)	
2501–5000	18	13.75 (9.60–21.30)	
>5000	14	20.24 (18.80–24.10)	
Diagnostic Method			
Neurologist evaluation	9	15.40 (11.30–23.40)	0.737
Neurological tests	62	18.90 (12.20–24.70)	
Neurologist evaluation and tests	8	21.30 (12.0–28.90)	
Region			
Europe	25	12.10 (9.94–23.90)	0.110
North America	13	20.10 (19.0–24.70)	
South America	3	34.0 (29.15–35.75)	
Asia	35	19.44 (13.25–25.55)	
Africa	2	25.70 (18.40–33.0)	
Australia	2	20.50 (7.70–33.30)	

Legend: median expressed in percentage.

**Table 2:** Summary of cognitive impairment incidence as reported by the 11 studies included.

	Studies	With Cognitive Impairment Median (25–75 Percentile)	*p* Value
Inclusion Age			
50–59	3	30.70 (26.35–36.24)	0.035 *
60–69	4	71.11 (58.44–145.98)	
≥70	4	58.45 (51.84–68.45)	
Participants Number			
<1001	2	51.09 (41.77–60.40)	0.693
1001–2500	5	56.50 (41.19–76.50)	
2501–5000	3	51.45 (41.08–58.44)	
>5000	1	215	
Diagnostic Method			
Neurologist evaluation	3	76.50 (59.14–145.75)	0.737
Neurological tests	5	51.45 (30.70–60.40)	
Neurologist evaluation and tests	3	56.50 (51.82–60.96)	
Region			
Europe	5	56.50 (51.45–76.50)	0.285
North America	5	60.40 (47.19–65.42)	
Asia	1	22	

(* significant at *p* < 0.05). Legend: median expressed in per 1000 person-years.

## Supplementary Materials

The following are available online at https://www.mdpi.com/2308-3417/5/4/84/s1, Supplemental Table S1: Cognitive Impairment Crude Prevalence, Supplemental Table S2: Cognitive Impairment Crude Incidence, Supplemental Table S3: Quality Assessment Tool for Observational Cohort and Cross-Sectional Studies; Supplemental Table S4: PRISMA 2009 checklist.
