# Supplementary material for: Global Cognitive Impairment Prevalence and Incidence in Community Dwelling Older Adults—A Systematic Review
Source: Geriatrics (Basel). 2020 Oct 27;5(4):84. doi: 10.3390/geriatrics5040084 (PMC7709591; doi:10.3390/geriatrics5040084)
Supplement: Supplementary file 1 [file geriatrics-05-00084-s001.zip › supplemental 2/Paper I - Supplemental 2 - Cognitive Impairment Crude Incidence.docx]

Supplemental Table 2 – Cognitive Impairment Crude Incidence

| World Region | Author, Year, Country | Incidence per 1000 person-year | Follow-up (years) | Sample size (baseline) | Age Cut-off | CI definition | Construct |
| --- | --- | --- | --- | --- | --- | --- | --- |
| Europe | Veronese N, 2016, Italy**^1^** | 51.5 | 4.4 | 2618 | >=65 | MMSE<24 |  |
| Europe | Luck T, 2010, Germany**^2^** | 56.5 (50.7–62.7) | 3 | 2331 | >75 | 1) No dementia; 2) Evidence of cognitive decline: self and/or informant report; 3) Preserved basic activities of daily living. | MCI |
| Europe | Etgen T, 2010, Germany**^3^** | 30.7 | 2 | 3903 | >55 | 6-Item Cognitive Impairment Test - scores higher than 7. | CI |
| Europe | Luck T, 2010, Germany**^4^** | 76.5 (64.7-90.4) | 8 | 1692 | >75 | 1) Absence of dementia; 2) Preserved ADLs or only minimal impairment in complex instrumental functions: assessed using the SIDAM-ADL scale; 3) Evidence of cognitive decline. | MCI |
| Europe | Ravaglia G, 2008, Italy**^5^** | 76.8 (66.8-88.4) | 4 | 1016 | >65 | Age- and education-adjusted score 1.5 SDs or fewer below the reference threshold on any of the tests used for detailed neuropsychological testing. | MCI |
| North America | Alhurani, 2016, USA**^7^** | 47.19 | 4.4 | 1895 | >=70 | ClinClinical dementia rating scale Functional activities questionnaire + neurological evaluation; Clinical dementia Rating Scale; Functional activities Questionnaire, neurological evaluation | MCI |
| North America | Potvin O, 2011, Canada**^8^** | 65.42 | 1 | 2785 | >65 | MMSE score below the 15th percentile according to norms for age, education, and sex | CIND |
| North America | Plassman BL, 2011, USA**^9^** | 60.4 (45.6-75.3) | 8 | 456 | >72 | 1) Memory complaint; 2) Below a 1.5-SD cut-off using normative corrections for age, years of education, race/ethnicity, and sex; 3) Preserved activities of daily living; 4) No diagnosis of dementia. | CIND |
| North America | Mejia-Arango S, 2011, Mexico**^10^** | 215 (205-224) | 2 | 7166 | >60 | 10 th percentile by sex and educational level (CCCE) or IQCODE | CIND |
| North America | Boyle PA, 2010, USA**^11^** | 41.77 | 12 | 761 | >54 | Word List Memory, Word List Recall, and Word List Recognition; semantic memory was assessed via three tests: a 15-item version of the Boston Naming Test, Verbal Fluency, and a 15-item reading test; working memory was assessed via three tests: Digit Span Forward, Digit Span Backward and Digit Ordering; perceptual speed was assessed via four tests: Symbol Digit Modalities Test, Number Comparison, and two indices from a modified version of the Stroop Neuropsychological Screening Test; and visuospatial abilities were assessed via two tests: a 15-item version of Judgment of Line Orientation and a 16-item version of Standard Progressive Matrices. One additional test, Complex Ideational Material | MCI |
| Asia | Feng L, 2016, Singapore**^12^** | 22 | 3 | 1575 | >=55 | MMSE<23 | CI |

REFERENCES

1. Veronese N, Stubbs B, Trevisan C, et al. What physical performance measures predict incident cognitive decline among intact older adults? A 4.4year follow up study. Exp Gerontol 2016;81:110-8.

2. Luck T, Luppa M, Briel S, Riedel-Heller SG. Incidence of mild cognitive impairment: a systematic review. Dement Geriatr Cogn Disord 2010;29:164-75.

3. Etgen T, Sander D, Huntgeburth U, Poppert H, Forstl H, Bickel H. Physical activity and incident cognitive impairment in elderly persons: the INVADE study. Arch Intern Med 2010;170:186-93.

4. Luck T, Luppa M, Briel S, et al. Mild cognitive impairment: incidence and risk factors: results of the leipzig longitudinal study of the aged. J Am Geriatr Soc 2010;58:1903-10.

5. Ravaglia G, Forti P, Montesi F, et al. Mild cognitive impairment: epidemiology and dementia risk in an elderly Italian population. J Am Geriatr Soc 2008;56:51-8.

6. Zanetti M, Ballabio C, Abbate C, Cutaia C, Vergani C, Bergamaschini L. Mild cognitive impairment subtypes and vascular dementia in community-dwelling elderly people: a 3-year follow-up study. J Am Geriatr Soc 2006;54:580-6.

7. Alhurani RE, Vassilaki M, Aakre JA, et al. Decline in Weight and Incident Mild Cognitive Impairment: Mayo Clinic Study of Aging. JAMA Neurol 2016;73:439-46.

8. Potvin O, Forget H, Grenier S, Preville M, Hudon C. Anxiety, depression, and 1-year incident cognitive impairment in community-dwelling older adults. J Am Geriatr Soc 2011;59:1421-8.

9. Plassman BL, Langa KM, McCammon RJ, et al. Incidence of dementia and cognitive impairment, not dementia in the United States. Ann Neurol 2011;70:418-26.

10. Mejia-Arango S, Gutierrez LM. Prevalence and incidence rates of dementia and cognitive impairment no dementia in the Mexican population: data from the Mexican Health and Aging Study. J Aging Health 2011;23:1050-74.

11. Boyle PA, Buchman AS, Wilson RS, Leurgans SE, Bennett DA. Physical frailty is associated with incident mild cognitive impairment in community-based older persons. J Am Geriatr Soc 2010;58:248-55.

12. Feng L, Nyunt MS, Gao Q, et al. Physical Frailty, Cognitive Impairment, and the Risk of Neurocognitive Disorder in the Singapore Longitudinal Ageing Studies. J Gerontol A Biol Sci Med Sci 2017;72:369-75.
