# Supplementary material for: Global Cognitive Impairment Prevalence and Incidence in Community Dwelling Older Adults—A Systematic Review
Source: Geriatrics (Basel). 2020 Oct 27;5(4):84. doi: 10.3390/geriatrics5040084 (PMC7709591; doi:10.3390/geriatrics5040084)
Supplement: Supplementary file 1 [file geriatrics-05-00084-s001.zip › supplemental 2/Paper I - Supplemental 1 - Cognitive Impairment Crude Prevalence.docx]

Supplemental Table 1 – Cognitive Impairment Crude Prevalence

| **Region** | **Author, Year, Country** | **Prevalence % (95% CI)** | **Sample size** | **Age Cut-off** | **CI definition** | **Construct** |
| --- | --- | --- | --- | --- | --- | --- |
| Europe | Janelidze, 2018, Georgia^1^ | 27,7 (22.2-33.9) | 238 | >=65 | MoCA >1.5 *SD* below mean value | MCI |
| Europe | Freak-Poli,2018, Holland^2^ | 9.33 (8.5-10.3) | 4201 | >=60 | MMSE<26 | MCI |
| Europe | Zaganas, 2018, Greece^3^ | 32.4(30.8-34.0) | 3140 | >=60 | MMSE<24 | MCI |
| Europe | Tsolaki, 2017, Greece^4^ | 15.3 (20-28.2) | 443 | >=61 | Neuropsychological battery of tests | MCI |
| Europe | Veronese N, 2016, Italy ^5^ | 32.7 (30.5-34.9) | 2618 | >=65 | MMSE<24 | CI |
| Europe | Wu YT, 2016, England^6^ | 33.7 (31.8-35.6) | 2424 | >=65 | MMSE<=25 | CI |
| Europe | Lara E, 2016, Spain^7^ | 9.6 (8.7-10,6) | 3625 | >50 | 1) Presence of cognitive concerns; 2) Objective evidence of impairment in one or more cognitive domains; 3) Preservation of independence in functional abilities; 4) No dementia. | MCI |
| Europe | Papachristou E, 2015, Great Britain^8^ | 41 (39.1-44.1) | 1530 | >76 | TYM, a simple 10-task self-assessment cognitive screening instrument which has sound psychometric properties scores between 33 and 45 (if older than 80 years of age) or 46 (if younger than 80 years of age) | MCI |
| Europe | Brujin RF, 2014, Holland^9^ | 9.94 (9.0-10.9) | 4198 | >55 | 1) Presence of subjective cognitive complaints; 2) Presence of objective cognitive impairment; 3) Absence of dementia. | MCI |
| Europe | Juncos-Rabadán O, 2014, Spain^10^ | 31.4 (27.9-35.0) | 689 | >50 | 1) Evidence of concern about a change in cognition, in comparison with the previous level; 2) Evidence of poorer performance in one or more cognitive domains that is greater than expected for the patient's age and educational background; 3) Preservation of independence in functional abilities; 4) Non-fulfilment of diagnostic criteria for dementia. | MCI |
| Europe | Moretti F, 2013, Italy^11^ | 24.5 (23.5-25.4) | 6921 | >61 | Score less than 1.5 SDs below the mean value scored by subjects of comparable age and education. | MCI |
| Europe | Rodríguez-Sánchez E, 2011, Spain^12^ | 14.9 (10.6-19.2) | 327 | >65 | 1) Mild cognitive or functional impairment reported by the participant or informant that did not meet criteria for dementia; 2) Performance on neuropsychological or functional measures that was both below expectations and ≥ 0.5 standard deviations below published norms on any test. | CI |
| Europe | Paul C, 2010, Portugal^13^ | 9.6 (7.7-11.0) | 1268 | >50 | Portuguese version MMSE adapted to different education levels and illiterate people | CI |
| Europe | Nunes B, 2010, Portugal^14^ | 12.0% (9.3-15.4) | 433 | 55-79 | Mini-Mental State Exam and neurological evaluation | CIND |
| Europe | Dlugaj M, 2010, Germany^15^ | 12.1 (9.8-14.4) | 4145 | >50 | 1) Objective memory disorder; 2) Absence of other cognitive disorders or repercussions on daily life; 3) Normal general cognitive function; 4) Absence of dementia. | MCI |
| Europe | Etgen T, 2010, Germany^16^ | 10.7 (9.8-11.7) | 3903 | >55 | 6-Item Cognitive Impairment Test - scores higher than 7 are consistent with cognitive impairment | CI |
| Europe | Luck T, 2010, Germany^17^ | 11.6 (10.3-13.0) | 2331 | >75 | 1) No dementia; 2) Evidence of cognitive decline: self and/or informant report; 3) Preserved basic activities of daily living. | MCI |
| Europe | Ravaglia G, 2008, Italy^18^ | 7.7 (6.1-9.7) | 1016 | >65 | MCI was defined as age- and education-adjusted score 1.5 SDs or fewer below the reference threshold on any of the tests used for detailed neuropsychological testing. | MCI |
| Europe | Luck T, 2007, Germany^19^ | 15.4 (14.1-16.6) | 3242 | >75 | 1) No dementia; 2) Evidence of cognitive decline; 3) Preserved basic activities of daily living. | MCI |
| Europe | Zanetti M, 2006, Italy^20^ | 16.2 (12.8-20.2) | 400 | >65 | MMSE <= 24, with age and education correction and the Clock Drawing Test. | MCI |
| Europe | De Ronchi D, 2005, Italy^21^ | 5.1 (4.6-5.6) | 7930 | >61 | Scored 2 or more standard deviations lower than the corrected mean MMSE score. | CIND |
| Europe | Hanninen, 2002, Finland^22^ | 5.3 (3.9-7.1) | 806 | >=60 | Score 1.5 SD below the cut-off | MCI |
| Europe | Saks K, 2001, Estonia^23^ | 23.1 (19.8-26.6) | 811 | >=65 | MMSE <25 | MCI |
| Europe | Frisoni GB, 2000, Sweden^24^ | 15 (13.1-16.9) | 1435 | 75-95 | 1) One standard deviation below the mean of age- and education-defined strata; 2) One standard deviation below the age- and education- specific mean computed from a statistical mode. | MCI |
| Europe | Di Carlo A, 2000, Italy^25^ | 10.7 (9.7-11.8) | 3425 | 65-84 | 1) MMSE<24; 2) Neurologist evaluation. | CIND |
| North America | Aliberti, 2018, USA^26^ | 19 | 7338 | >=65 | Aproach for HRS self-respondents.16,17 The method includes the following cognitive tests: (1) immediate and delayed recall of 10 com- mon nouns, (2) serial subtractions by 7, and (3) a backward count task from 20. | CIND |
| North America | Richard E, 2013, USA^27^ | 19.9 (18.2-21.6) | 2160 | >65 | Neurologist evaluation. | MCI |
| North America | Ortiz GG, 2012, Mexico^28^ | 13.8 (11.9–16.0) | 1142 | >60 | MMSE score <18 | CI |
| North America | Mejia-Arango S, 2011, Mexico^29^ | 25.1 (23.1-26.3) | 7166 | >60 | 10^th^ percentile of Cross-Cultural Cognitive Examination or IQCODE | CIND |
| North America | Cortés AR, 2011, Mexico^30^ | 24.7 (20.1-29.8) | 324 | >60 | MMSE≤24 | CI |
| North America | Gamaldo AA, 2011, USA^31^ | 22.4 (19.0-26.1) | 554 | >50 | MMSE and SPQMSQ score 1.5 SD below the sample mean. | MCI |
| North America | Sachs GA, 2011, USA^32^ | 20.1 (19.0-21.5) | 3957 | >60 | Short Portable Mental Status Questionnaire | CI |
| North America | Langa KM, 2008, USA^33^ | 12.2 | 7486 | >70 | 35-point scale that includes: an immediate and delayed 10-noun free recall test to measure memory; a serial seven subtraction test to measure working memory; a counting backwards test to measure speed of mental processing; an object naming test to measure knowledge and language; and recall of the date, the president, and the vice-president to measure orientation. Prevalence adjusted with HRS survey. | CI |
| North America | Mejia-Arango S, 2007, Mexico^34^ | 7.1 (6.3-7.8) | 4183 | >65 | 10^th^ percentile of Cross-Cultural Cognitive Examination. | CI |
| North America | Manly JJ, 2005, USA^35^ | 28.3 (25.9-30.8) | 1315 | >65 | 1) Memory complaint; 2) Score below a 1.5-SD cut-off using normative corrections for age, years of education, race/ethnicity, and sex; 3) Preserved activities of daily living; 4) No diagnosis of dementia. | MCI |
| North America | Purser JL, 2005, USA^36^ | 24.7 (23.2-26.2) | 3673 | >65 | 0 to 3 errors indicating intact cognition, and 4 or more errors indicating impaired cognition. | MCI |
| North America | Lopez OL, 2003, USA^37^ | 22 (20.6-23.4) | 3602 | >=75 | Neurological, neuropsychological, neuroradiological and psychiatric testing. | MCI |
| North America | Lopez OL, 2003, USA^38^ | 19.0 (17.3-20.4) | 3608 | >75 | 1) Participants or their families reported cognitive problems; 2) There were no neurological, psychiatric, or systemic illnesses that could explain their presence of cognitive deficits. | MCI |
| South America | Pozo, 2018, Ecuador^39^ | 37,5 (29.6-45.9) | 144 | >=65 | MMSE<24 | CI |
| South America | Ono, 2018, Brasil^40^ | 24.3 (20.0-29.1) | 1702 | >=60 | MMSE<19 for no education or <23 for some education | CI |
| South America | Winter Holz A, 2013, Brasil^41^ | 34.0 (31.7-36.5) | 1514 | >60 | MMSE score <23 | CI |
| Asia | Han, 2018, South Korea^42^ | 27 (25.9-28) | 6818 | >=60 |  | MCI |
| Asia | Soleimani, 2018, Iran^43^ | 37 (32.1-41,9) | 393 | >=60 | MMSE<24 | MCI |
| Asia | Zhang Y, 2018, China^44^ | 23.3 (22.2-24.4) | 5558 | >=60 |  | CIND |
| Asia | Liu, 2018, China^45^ | 34.1(30.4-38.0) | 622 | >=65 | A cut-off score of 2 or greater on the AD8 | MCI |
| Asia | Rao D, 2017, China^46^ | 14.2(12.7-15.7) | 2111 | >=65 | (1) Cognitive concern or complaint by the subject or a person familiar with the subject, with a CDR score of 0.5; (2) objective impairment in one or more cognitive domain (memory, executive function, visuo-constructive skills, or verbal fluency), based on perfor- mance 1.5 standard deviation below that expected for the subject’s age and education; (3) essentially normal functional activity, based on the results of the CDR and FAQ; and (4) absence of dementia, based on the Diagnostic and Statistical Manual of Mental Disorders | MCI |
| Asia | Pedraza, 2017, India^47^ | 34 (31.2-36.5) | 1235 | >=50 | MOCA and neurologists evaluation | MCI |
| Asia | Ren, 2017, China^48^ | 29,9(25.9-34.3) | 480 | >80 | MMSE + MOCA | MCI |
| Asia | Han, 2017, China^49^ | 13.7 (12,1-15,2) | 2017 | >=60 | The thresholds for those who were illiterate, or attended at most primary school, middle school, or university were ≤ 17, 17–20, 21–22, and 23–24 | MCI |
| Asia | Feng L, 2016, Singapore^50^ | 9.0 (7.8-10.8) | 1575 | >=55 | MMSE<23 | CI |
| Asia | Tang HD, 2016, China^51^ | 6.5 (5.7-7.4) | 3471 | >50 | education modified MMSE score | MCI |
| Asia | Tzivian L, 2016, Philippines^52^ | 28.9 (26.9-30.9) | 2050 | >50 | 1) Presence of a subjective cognitive complaint (participants were asked if their cognitive performance had changed during the past 2 years. A complaint was considered present if the participant reported a decline in cognitive performance over time); 2) Presence of an objective cognitive impairment; 3) Not fulfil criteria for dementia (Diagnostic and Statistical Manual of Mental Disorders, DSM-IV); 4) Generally intact activities of daily living. | MCI |
| Asia | Vanoh D, 2016, Malaysia^53^ | 16 (14.1-17.7) | 1993 | >=60 | MMSE<23 | MCI |
| Asia | Nakamura, 2016, Japan^54^ | 8.4 (5.1-12.9) | 239 | >=65 | Hasegawa's dementia scale (HDS-R) was used to assess cognitive function, and cognitive impairment was defined as a HDS-R score ≤20 | MCI |
| Asia | Ma F, 2016, China^55^ | 11.3(8.1-14.4) | 5214 | >=65 | Neuropsychological assessments and clinical examinations | MCI |
| Asia | Shimada H, 2016, Japan^56^ | 17.4 (16.2-18.7) | 4290 | >65 | 1) Objective cognitive impairment (indicated by an age- and education-adjusted score of at least 1.5 standard deviations below the reference threshold on tests commonly used for detailed neuropsychological assessment); 2) No evidence of functional dependency (e.g., no need for supervision or external assistance in performing ADL); 3) Exclusion by the clinical criteria for dementia. | MCI |
| Asia | Lyu J, 2016, South Korea^57^ | 28.5 (26.0-31.1) | 1759 | >65 | Based on age, gender, and educational strata, the cut- off scores (2 standard deviation of the mean) from the K- MMSE normative data were used to determine cognitive im- pairment | CI |
| Asia | Giri M, 2016, China^58^ | 12.6 (10.0–15.8) | 538 | >60 | Cognitive concern or complaint by the subject or a person familiar with the subject, with a CDR score of 0.5; (2) objective impairment in one or more cognitive domain (memory, executive function, visuo-constructive skills, or verbal fluency), based on performance 1.5 standard deviation below that expected for the subject’s age and education; (3) essentially normal functional activity, based on the results of the CDR and FAQ; and (4) absence of dementia, based on the Diagnostic and Statistical Manual of Mental Disorders. | CI |
| Asia | Liu M, 2015, China^59^ | 15.9 (14.4-17.5) | 2102 | >60 | MMSE - < 17 for illiteracy participants; <20 for participants with 1–6 education years; <24 for participants with more than 6 education years. | MCI |
| Asia | Sun Y, 2014, Taiwan^60^ | 18.8 (17.9–19.6) | 10432 | >=65 | Score <=24 in literate elders and <=13 in illiterate elders in Taiwanese Mental State Examination (TMSE) | MCI |
| Asia | Xu S, 2014, China^61^ | 21.3 (19.7-22.9) | 2601 | >60 | MOCA + neurologistis evaluation | MCI |
| Asia | Su X, 2014, China^62^ | 18.5 (15.8-21.8) | 815 | >60 | 1) Intact ADL; 2) Memory complaints (either self-reported or family members, caregivers); 3) MMSE; 4) Essentially intact ADL and IADL; 5) No clear dementia; 6) No abnormal memory impairment for age. | MCI |
| Asia | Zhang Y, 2014, China^63^ | 23.3 (22.2-24.4) | 5550 | >60 | 1) Mild cognitive or functional impairment that did not meet the criteria for dementia; 2) Performance on neuropsychological or functional measures below expectations and ≥ 0.5 standard deviations below published norms on any test. | CIND |
| Asia | Jia J, 2014, China^64^ | 20.8 (20.0–21.6), | 10276 | >65 | 1) Scored at least 1.5 standard deviations below the norm in memory, executive function, language, or visuoconstructive skill; 2) Global CDR score of 0.5 or less; 3) Preserved ability to perform daily activities and social functions; 4) Absence of dementia. | MCI |
| Asia | Leggett A, 2013, Vietnam^65^ | 12.9 (10.0-16.2) | 489 | >55 | MMSE<24 (education adjusted) | CI |
| Asia | Shimada H, 2013, Japan^66^ | 18.8 (17.7-19.8) | 5104 | >65 | MMSE | MCI |
| Asia | Zhu YP, 2013, China^67^ | 20.7 (18.5-23.1) | 1211 | >60 | MMSE and MOCA | MCI |
| Asia | Rashid AK, 2012, Malaysia^68^ | 11.0 (8.2-14.4) | 418 | >60 | Elderly Cognitive Assessment Questionnaire (ECAQ) - A score of 7 or more is indicative of normal memory and score of 4 and below indicates probable dementia. | CI |
| Asia | Lee LK, 2012, Malaysia^69^ | 21.1 (16.8-25.8) | 333 | >60 | MMSE and geriatrician, gerontologist and clinical psychologist evaluations | MCI |
| Asia | Wada-Isoe K, 2012, Japan^70^ | 23.4 (20.7–26.2) | 900 | >65 | 1) Cognitive complaints; 2) Evidence of decline in cognitive function; 3) No impairment of functional activities of daily living; 4) No dementia. | MCI |
| Asia | Zhuang JP, 2012, China^71^ | 8.4 (8.3-8.5) | 3176 | >=55 | MMSE score ≤ 17 for illiterates;  ≤ 20 for primary school graduates (≥6 years of education); ≤ 24 for junior school graduates or above (≥9 years of education). | CI |
| Asia | Kim KW, 2011, South Korea^72^ | 24.1 (21.0–27.2) | 8199 | >65 | MMSE and CERAD-K Neuropsychological Assesment Battery | MCI |
| Asia | Lu J, 2011, China^73^ | 20.4 (19.3-21.5) | 8411 | >65 | MOCA | CI |
| Asia | Yen CH, 2010, Taiwan^74^ | 9.9 (8.5-11.5) | 1626 | >60 | Nine item Short Portable Mental Status Questionnaire - 4 errors or more in 9 possible | CI |
| Asia | Choo IH, 2009, South Korea^75^ | 31.9 (28.0–35.8) | 643 | >65 | 1) A global CDR (clinical dementia rating- index of 0.5; 2) Exclusion of dementia | CIND |
| Asia | Taboonpong, 2008, Thailand^76^ | 15.0 (11.7-18.8) | 420 | >60 | Chula Mental Test (CMT)<14 in 0-19 - abnormal cognition | CI |
| Africa | Ogunniyi A, 2016, Nigeria^77^ | 18.4 (14.9–21.8) | 613 | >65 | IDEA cognitive screen. It tested 10‐word learning (repeated three times), orientation, verbal fluency, abstract reasoning, delayed recall and praxis. For cut‐off score of ≤7 | CI |
| Africa | Inzelberg R, 2015, Israel^78^ | 33 (29.7-35.9) | 906 | >=65 | Neurological and cognitive examinations | MCI |
| Australia | Anderson TM, 2007, Australia^79^ | 7.7 (6.5-9.0) | 1792 | >65 | MMSE≤23 | CI |
| Australia | Low LF, 2004, Australia^80^ | 33.3 (25.6-42.4) | 131 | 70-79 | 1.5 SDs below age-corrected (and education where available) norms on at least one test in the neuropsychological battery and MMSE <24 | CIND |

REFERENCES

1. Janelidze M, Mikeladze N, Bochorishvili N, et al. Mild Cognitive Impairment in Republic of Georgia. Gerontology & geriatric medicine 2018;4:2333721418771408.

2. Freak-Poli R, Licher S, Ryan J, Ikram MA, Tiemeier H. Cognitive Impairment, Sexual Activity and Physical Tenderness in Community-Dwelling Older Adults: A Cross-Sectional Exploration. Gerontology 2018;64:589-602.

3. Zaganas IV, Simos P, Basta M, et al. The Cretan Aging Cohort: Cohort Description and Burden of Dementia and Mild Cognitive Impairment. American journal of Alzheimer's disease and other dementias 2019;34:23-33.

4. Tsolaki M, Gkioka M, Verykouki E, Galoutzi N, Kavalou E, Pattakou-Parasyri V. Prevalence of Dementia, Depression, and Mild Cognitive Impairment in a Rural Area of the Island of Crete, Greece. American journal of Alzheimer's disease and other dementias 2017;32:252-64.

5. Veronese N, Stubbs B, Trevisan C, et al. What physical performance measures predict incident cognitive decline among intact older adults? A 4.4year follow up study. Experimental gerontology 2016;81:110-8.

6. Wu YT, Prina AM, Jones AP, Barnes LE, Matthews FE, Brayne C. Community environment, cognitive impairment and dementia in later life: results from the Cognitive Function and Ageing Study. Age and ageing 2015;44:1005-11.

7. Lara E, Koyanagi A, Olaya B, et al. Mild cognitive impairment in a Spanish representative sample: prevalence and associated factors. International journal of geriatric psychiatry 2016;31:858-67.

8. Papachristou E, Ramsay SE, Lennon LT, et al. The relationships between body composition characteristics and cognitive functioning in a population-based sample of older British men. BMC geriatrics 2015;15:172.

9. de Bruijn RF, Akoudad S, Cremers LG, et al. Determinants, MRI correlates, and prognosis of mild cognitive impairment: the Rotterdam Study. Journal of Alzheimer's disease : JAD 2014;42 Suppl 3:S239-49.

10. Juncos-Rabadan O, Pereiro AX, Facal D, et al. Prevalence and correlates of mild cognitive impairment in adults aged over 50 years with subjective cognitive complaints in primary care centers. Geriatrics & gerontology international 2014;14:667-73.

11. Moretti F, De Ronchi D, Palmer K, et al. Prevalence and characteristics of mild cognitive impairment in the general population. Data from an Italian population-based study: The Faenza Project. Aging & mental health 2013;17:267-75.

12. Rodriguez-Sanchez E, Mora-Simon S, Patino-Alonso MC, et al. Prevalence of cognitive impairment in individuals aged over 65 in an urban area: DERIVA study. BMC neurology 2011;11:147.

13. Paul C, Ribeiro O, Santos P. Cognitive impairment in old people living in the community. Archives of gerontology and geriatrics 2010;51:121-4.

14. Nunes B, Silva RD, Cruz VT, Roriz JM, Pais J, Silva MC. Prevalence and pattern of cognitive impairment in rural and urban populations from Northern Portugal. BMC neurology 2010;10:42.

15. Dlugaj M, Weimar C, Wege N, et al. Prevalence of mild cognitive impairment and its subtypes in the Heinz Nixdorf Recall study cohort. Dementia and geriatric cognitive disorders 2010;30:362-73.

16. Etgen T, Sander D, Huntgeburth U, Poppert H, Forstl H, Bickel H. Physical activity and incident cognitive impairment in elderly persons: the INVADE study. Archives of internal medicine 2010;170:186-93.

17. Luck T, Riedel-Heller SG, Luppa M, et al. Risk factors for incident mild cognitive impairment--results from the German Study on Ageing, Cognition and Dementia in Primary Care Patients (AgeCoDe). Acta psychiatrica Scandinavica 2010;121:260-72.

18. Ravaglia G, Forti P, Montesi F, et al. Mild cognitive impairment: epidemiology and dementia risk in an elderly Italian population. Journal of the American Geriatrics Society 2008;56:51-8.

19. Luck T, Riedel-Heller SG, Kaduszkiewicz H, et al. Mild cognitive impairment in general practice: age-specific prevalence and correlate results from the German study on ageing, cognition and dementia in primary care patients (AgeCoDe). Dementia and geriatric cognitive disorders 2007;24:307-16.

20. Zanetti M, Ballabio C, Abbate C, Cutaia C, Vergani C, Bergamaschini L. Mild cognitive impairment subtypes and vascular dementia in community-dwelling elderly people: a 3-year follow-up study. Journal of the American Geriatrics Society 2006;54:580-6.

21. De Ronchi D, Berardi D, Menchetti M, et al. Occurrence of cognitive impairment and dementia after the age of 60: a population-based study from Northern Italy. Dementia and geriatric cognitive disorders 2005;19:97-105.

22. Hanninen T, Hallikainen M, Tuomainen S, Vanhanen M, Soininen H. Prevalence of mild cognitive impairment: a population-based study in elderly subjects. Acta neurologica Scandinavica 2002;106:148-54.

23. Saks K, Kolk H, Allev R, et al. Health status of the older population in Estonia. Croatian medical journal 2001;42:663-8.

24. Frisoni GB, Fratiglioni L, Fastbom J, Guo Z, Viitanen M, Winblad B. Mild cognitive impairment in the population and physical health: data on 1,435 individuals aged 75 to 95. The journals of gerontology Series A, Biological sciences and medical sciences 2000;55:M322-8.

25. Di Carlo A, Baldereschi M, Amaducci L, et al. Cognitive impairment without dementia in older people: prevalence, vascular risk factors, impact on disability. The Italian Longitudinal Study on Aging. Journal of the American Geriatrics Society 2000;48:775-82.

26. Aliberti MJR, Cenzer IS, Smith AK, Lee SJ, Yaffe K, Covinsky KE. Assessing Risk for Adverse Outcomes in Older Adults: The Need to Include Both Physical Frailty and Cognition. Journal of the American Geriatrics Society 2018.

27. Richard E, Reitz C, Honig LH, et al. Late-life depression, mild cognitive impairment, and dementia. JAMA neurology 2013;70:374-82.

28. Ortiz GG, Arias-Merino ED, Flores-Saiffe ME, Velazquez-Brizuela IE, Macias-Islas MA, Pacheco-Moises FP. Prevalence of Cognitive Impairment and Depression among a Population Aged over 60 Years in the Metropolitan Area of Guadalajara, Mexico. Current gerontology and geriatrics research 2012;2012:175019.

29. Mejia-Arango S, Gutierrez LM. Prevalence and incidence rates of dementia and cognitive impairment no dementia in the Mexican population: data from the Mexican Health and Aging Study. Journal of aging and health 2011;23:1050-74.

30. Cortes AR, Villarreal E, Galicia L, Martinez L, Vargas ER. [Cross sectional geriatric assessment of Mexican older people]. Revista medica de Chile 2011;139:725-31.

31. Gamaldo AA, Allaire JC, Sims RC, Whitfield KE. Assessing mild cognitive impairment among older African Americans. International journal of geriatric psychiatry 2010;25:748-55.

32. Sachs-Erisson N, Sawyer K, Corsentino E, Collins N, Steffens DC. The moderating effect of the APOE [small element of] 4 allele on the relationship between hippocampal volume and cognitive decline in older depressed patients. The American journal of geriatric psychiatry : official journal of the American Association for Geriatric Psychiatry 2011;19:23-32.

33. Langa KM, Larson EB, Karlawish JH, et al. Trends in the prevalence and mortality of cognitive impairment in the United States: is there evidence of a compression of cognitive morbidity? Alzheimer's & dementia : the journal of the Alzheimer's Association 2008;4:134-44.

34. Mejia-Arango S, Miguel-Jaimes A, Villa A, Ruiz-Arregui L, Gutierrez-Robledo LM. [Cognitive impairment and associated factors in older adults in Mexico]. Salud publica de Mexico 2007;49 Suppl 4:S475-81.

35. Manly JJ, Bell-McGinty S, Tang MX, Schupf N, Stern Y, Mayeux R. Implementing diagnostic criteria and estimating frequency of mild cognitive impairment in an urban community. Archives of neurology 2005;62:1739-46.

36. Purser JL, Fillenbaum GG, Pieper CF, Wallace RB. Mild cognitive impairment and 10-year trajectories of disability in the Iowa Established Populations for Epidemiologic Studies of the Elderly cohort. Journal of the American Geriatrics Society 2005;53:1966-72.

37. Lopez OL. [Classification of mild cognitive impairment in a population study]. Revista de neurologia 2003;37:140-4.

38. Lopez OL, Jagust WJ, DeKosky ST, et al. Prevalence and classification of mild cognitive impairment in the Cardiovascular Health Study Cognition Study: part 1. Archives of neurology 2003;60:1385-9.

39. Espinosa Del Pozo PH, Espinosa PS, Donadi EA, et al. Cognitive Decline in Adults Aged 65 and Older in Cumbaya, Quito, Ecuador: Prevalence and Risk Factors. Cureus 2018;10:e3269.

40. Ono LM, Confortin SC, Figueiro TH, Rech CR, d'Orsi E. Influence of instrumental activities of daily living on the cognitive impairment: EpiFloripa study. Aging & mental health 2018:1-5.

41. Winter Holz A, Nunes BP, Thume E, Lange C, Facchini LA. Prevalence of cognitive impairment and associated factors among the elderly in Bage, Rio Grande do Sul, Brazil. Revista brasileira de epidemiologia = Brazilian journal of epidemiology 2013;16:880-8.

42. Han JW, Kim TH, Kwak KP, et al. Overview of the Korean Longitudinal Study on Cognitive Aging and Dementia. Psychiatry investigation 2018;15:767-74.

43. Soleimani R, Shokrgozar S, Fallahi M, Kafi H, Kiani M. An investigation into the prevalence of cognitive impairment and the performance of older adults in Guilan province. Journal of medicine and life 2018;11:247-53.

44. Zhang Y, Guan Y, Shi Z, et al. Sex Differences in the Prevalence of and Risk Factors for Cognitive Impairment No Dementia among the Elderly in a Rural Area of Northern China: A Population-Based Cross-Sectional Study. Neuroepidemiology 2018;52:25-31.

45. Liu X, Yin X, Tan A, et al. Correlates of Mild Cognitive Impairment of Community-Dwelling Older Adults in Wuhan, China. International journal of environmental research and public health 2018;15.

46. Rao D, Luo X, Tang M, et al. Prevalence of mild cognitive impairment and its subtypes in community-dwelling residents aged 65 years or older in Guangzhou, China. Archives of gerontology and geriatrics 2018;75:70-5.

47. Pedraza OL, Montes AMS, Sierra FA, et al. Mild cognitive impairment (MCI) and dementia in a sample of adults in the city of Bogota. Dementia & neuropsychologia 2017;11:262-9.

48. Ren L, Zheng Y, Wu L, et al. Investigation of the prevalence of Cognitive Impairment and its risk factors within the elderly population in Shanghai, China. Scientific reports 2018;8:3575.

49. Han R, Tang Z, Ma L. Related factors of cognitive impairment in community-dwelling older adults in Beijing Longitudinal Study of Aging. Aging clinical and experimental research 2018.

50. Feng L, Nyunt MS, Gao Q, et al. Physical Frailty, Cognitive Impairment, and the Risk of Neurocognitive Disorder in the Singapore Longitudinal Ageing Studies. The journals of gerontology Series A, Biological sciences and medical sciences 2017;72:369-75.

51. Tang HD, Zhou Y, Gao X, et al. Prevalence and Risk Factor of Cognitive Impairment were Different between Urban and Rural Population: A Community-Based Study. Journal of Alzheimer's disease : JAD 2016;49:917-25.

52. Tzivian L, Dlugaj M, Winkler A, et al. Long-Term Air Pollution and Traffic Noise Exposures and Mild Cognitive Impairment in Older Adults: A Cross-Sectional Analysis of the Heinz Nixdorf Recall Study. Environmental health perspectives 2016;124:1361-8.

53. Vanoh D, Shahar S, Din NC, et al. Predictors of poor cognitive status among older Malaysian adults: baseline findings from the LRGS TUA cohort study. Aging clinical and experimental research 2017;29:173-82.

54. Nakamura K, Kitamura K, Watanabe Y, Shinoda H, Sato H, Someya T. Rural-urban differences in the prevalence of cognitive impairment in independent community-dwelling elderly residents of Ojiya city, Niigata Prefecture, Japan. Environmental health and preventive medicine 2016;21:422-9.

55. Ma F, Wu T, Zhao J, et al. Prevalence of Mild Cognitive Impairment and Its Subtypes among Chinese Older Adults: Role of Vascular Risk Factors. Dementia and geriatric cognitive disorders 2016;41:261-72.

56. Shimada H, Makizako H, Doi T, Tsutsumimoto K, Lee S, Suzuki T. Cognitive Impairment and Disability in Older Japanese Adults. PloS one 2016;11:e0158720.

57. Lyu J, Kim HY. Gender-Specific Incidence and Predictors of Cognitive Impairment among Older Koreans: Findings from a 6-Year Prospective Cohort Study. Psychiatry investigation 2016;13:473-9.

58. Giri M, Chen T, Yu W, Lu Y. Prevalence and correlates of cognitive impairment and depression among elderly people in the world's fastest growing city, Chongqing, People's Republic of China. Clinical interventions in aging 2016;11:1091-8.

59. Liu M, He Y, Jiang B, et al. Association between metabolic syndrome and mild cognitive impairment and its age difference in a Chinese community elderly population. Clinical endocrinology 2015;82:844-53.

60. Sun Y, Lee HJ, Yang SC, et al. A nationwide survey of mild cognitive impairment and dementia, including very mild dementia, in Taiwan. PloS one 2014;9:e100303.

61. Xu S, Xie B, Song M, et al. High prevalence of mild cognitive impairment in the elderly: a community-based study in four cities of the hebei province, china. Neuroepidemiology 2014;42:123-30.

62. Su X, Shang L, Xu Q, et al. Prevalence and predictors of mild cognitive impairment in Xi'an: a community-based study among the elders. PloS one 2014;9:e83217.

63. Zhang Y, Shi Z, Liu M, et al. Prevalence of cognitive impairment no dementia in a rural area of Northern China. Neuroepidemiology 2014;42:197-203.

64. Jia J, Zhou A, Wei C, et al. The prevalence of mild cognitive impairment and its etiological subtypes in elderly Chinese. Alzheimer's & dementia : the journal of the Alzheimer's Association 2014;10:439-47.

65. Leggett A, Zarit SH, Hoang CN, Nguyen HT. Correlates of cognitive impairment in older Vietnamese. Aging & mental health 2013;17:915-23.

66. Shimada H, Makizako H, Doi T, et al. Combined prevalence of frailty and mild cognitive impairment in a population of elderly Japanese people. Journal of the American Medical Directors Association 2013;14:518-24.

67. Zhu YP, Chen MF, Shen BH. [A prevalence study on mild cognitive impairment among elderly populations in Zhejiang province]. Zhonghua liu xing bing xue za zhi = Zhonghua liuxingbingxue zazhi 2013;34:475-7.

68. Rashid AK, Azizah AM, Rohana S. Cognitive impairment among the elderly Malays living in rural Malaysia. The Medical journal of Malaysia 2012;67:186-9.

69. Lee LK, Shahar S, Chin AV, Mohd Yusoff NA, Rajab N, Aziz SA. Prevalence of gender disparities and predictors affecting the occurrence of mild cognitive impairment (MCI). Archives of gerontology and geriatrics 2012;54:185-91.

70. Wada-Isoe K, Uemura Y, Nakashita S, et al. Prevalence of Dementia and Mild Cognitive Impairment in the Rural Island Town of Ama-cho, Japan. Dementia and geriatric cognitive disorders extra 2012;2:190-9.

71. Zhuang JP, Wang G, Cheng Q, et al. Cognitive impairment and the associated risk factors among the elderly in the Shanghai urban area: a pilot study from China. Translational neurodegeneration 2012;1:22.

72. Kim KW, Park JH, Kim MH, et al. A nationwide survey on the prevalence of dementia and mild cognitive impairment in South Korea. Journal of Alzheimer's disease : JAD 2011;23:281-91.

73. Lu J, Li D, Li F, et al. Montreal cognitive assessment in detecting cognitive impairment in Chinese elderly individuals: a population-based study. Journal of geriatric psychiatry and neurology 2011;24:184-90.

74. Yen CH, Yeh CJ, Wang CC, et al. Determinants of cognitive impairment over time among the elderly in Taiwan: results of the national longitudinal study. Archives of gerontology and geriatrics 2010;50 Suppl 1:S53-7.

75. Choo IH, Lee DY, Lee JH, et al. The prevalence of cognitive impairment with no dementia in older people: the Seoul study. International journal of geriatric psychiatry 2009;24:306-12.

76. Taboonpong S, Chailungka P, Aassanangkornchai S. Factors related to cognitive status among elders in southern Thailand. Nursing & health sciences 2008;10:188-94.

77. Ogunniyi A, Adebiyi AO, Adediran AB, Olakehinde OO, Siwoku AA. Prevalence estimates of major neurocognitive disorders in a rural Nigerian community. Brain and behavior 2016;6:e00481.

78. Inzelberg R, Massarwa M, Schechtman E, Strugatsky R, Farrer LA, Friedland RP. Estimating the risk for conversion from mild cognitive impairment to Alzheimer's disease in an elderly Arab community. Journal of Alzheimer's disease : JAD 2015;45:865-71.

79. Anderson TM, Sachdev PS, Brodaty H, Trollor JN, Andrews G. Effects of sociodemographic and health variables on Mini-Mental State Exam scores in older Australians. The American journal of geriatric psychiatry : official journal of the American Association for Geriatric Psychiatry 2007;15:467-76.

80. Low LF, Brodaty H, Edwards R, et al. The prevalence of "cognitive impairment no dementia" in community-dwelling elderly: a pilot study. The Australian and New Zealand journal of psychiatry 2004;38:725-31.
